# Surgery

**Published:** 1912-03

**Authors:** E. W. Hey Groves


### SURGERY.

**The surgery of gastric ulcers — Pathology.** — (1) The circulation in the stomach is so richly provided with blood-vessels in the sub-mucosa that it is possible to ligature four-fifths of the large vessels without causing any necrotic lesion; but on the other hand any agent which can cause an obliteration of a large area of the small vessels will produce a chronic indolent ulcer. Thus these have been experimentally produced by the injection of formalin, alcohol, hot salt solutions, and solutions of adrenalin extract into the gastric vessels (Payr, Benecke, Rosenbach). It is probable that an analogous condition of sclerosis of the small vessels, with an obliterative endarteritis, occurs as an antecedent causative condition in many clinical cases of ulceration. Spasms also may temporarily obliterate the lumen of the vessels and so predispose to ulceration. Thrombosis and retrograde portal embolism account for those cases of gastric ulcer which occur as a complication of septic lesions, such as appendicitis. (2) Nerve lesions. Division of the vagi nerves causes chronic ulceration of a variable character and extent (Zironi, Marchetti), but it is highly problematical whether this has any clinical significance. (3) Chemical changes. The increase in hydrochloric acid and in peptic activity seen in cases of gastric ulcer may be either the cause or the effect of the latter. For an ulcer can itself stimulate secretion, and by causing either pyloric stenosis or spasm, it can bring about a prolonged action of the gastric secretion upon the stomach walls. All these changes quickly disappear after the excision of the ulcer, as well as after a gastro-enterostomy, and this fact suggests rather that the ulcer is the primary condition. (4) Trauma and bacterial infection are seldom the sole cause of ulceration, but they may act as contributory causes when the predisposition to this lesion already exists. (5) Toxic conditions, *e.g.* acute appendicitis, may cause severe bleeding from the stomach, but usually this is more of the nature of a general weeping than an ulceration; or such conditions, including severe burns, may act either as specific gastric poisons, or as agents which act by causing thrombosis. Bolton has shown how the injection of emulsions of tissue cells, especially those from the stomach itself, may cause gastric ulcer. (6) Anæmia acting alone will not cause gastric ulcer, but when it occurs in association with other more directly potent agencies, it greatly retards the healing of these lesions.

**The relation to cancer.**—There has perhaps been no more important recent advance in the pathology of gastric disease than the proof that a large proportion of cases of gastric cancer begin as simple ulcers. All recent workers agree that this proportion is a large one, but about the exact figure there is great difference of opinion. Thus Payr, Küttner, Wilson and McCarty, Mayo Robson and W. Mayo give figures which vary from 25 to 71 per cent. These results have been discredited by those who regard the matter from a purely clinical standpoint. That is to say, there is only a small proportion of those who are known to have suffered from chronic ulcer who subsequently develop cancer. But if all cases of cancer are examined microscopically by serial sections, then it is proved that the majority are malignant developments of simple ulceration. It is easy to explain this discrepancy. The cases of cancer may have had a period of latent ulceration which was overlooked clinically, and this supplies a most important reason for submitting all cases of gastric disease to operative interference before sufficient time has elapsed to allow of malignant development.

**Symptoms.**—Pain is perhaps the most constant symptom of gastric ulcer, and its position corresponds roughly to that of the causative lesion. Thus, pain to the right of the epigastrium is due to a pyloric ulcer, that to the left is caused by an ulcer of the lesser curve, whilst pain in the back is produced by an ulcer adherent to the pancreas; and yet the gastric mucous membrane is quite insensitive to ordinary pain-producing stimuli. This has often been observed in operations done under local anæsthesia, and it has been fully elaborated by Hertz. According to this author, gastric pain is of twofold origin, either being caused by inflammatory adhesions dragging upon the sensitive parietal peritoneum, or by an excessive or unnatural spasm of its own fibres.

**Diagnosis.**—It has for long been recognised that the majority of chronic ulcers of the stomach cause bleeding, which can only be recognised by microscopical or chemical examination of the gastric contents or fæces. In about three-fourths of all cases occult bleeding thus occurs. Einhorn has introduced a simple and ingenious test and demonstration of the existence and situation of this bleeding. A small hollow weight, shaped like a little bucket, is attached to a long stout silk thread, and the patient swallows it like a pill, the end of the thread being securely fastened to one of the teeth. It is left overnight in the fasting stomach, and withdrawn next morning. If an ulcer exists, its position, *i.e.* its distance from the teeth, is marked on the white silk by an indelible blood-stain, and in some cases two stains have been present, and subsequent operation has revealed two ulcers.

In cases of severe hemorrhage from the stomach which do not yield to medicinal treatment but require operation, the bleeding often occurs from one or two quite small erosions. Rovsing has employed a quick and efficacious method of finding such bleeding points. He exposes the stomach, and then inserts through a small hole in its wall an endoscope with an air-dilating attachment. When the stomach is inflated and illuminated, the bleeding point or points show up as distinct shadows. A great number of gastroscopes have been introduced, of which by far the simplest, and therefore of most use for the uninitiated, is a simple straight tube, with the lamp at the distal extremity. But at present it seems likely that the use of the X-rays will quite supersede the various forms of endoscopic examination. The writings of Holzknecht are those in which most information can be found on this subject, and Schmieden has quite recently contributed a profusely illustrated paper upon it, which is followed by a useful list of other references. These authors hold that the X-rays, after feeding the patient with bismuth emulsion, are capable of demonstrating the size, shape, and position of the stomach, together with any abnormalities in the outline of its cavity, whether caused by ulcer, tumour, or hour-glass contraction ; but a very long experience of this method is necessary before its results can properly be interpreted, and we think that it is going too far to say that it makes exploratory operations quite unnecessary. Rowlands has pointed out that it is very easy to be deceived by the bismuth test and the X-rays, in mistaking a dilated and downwardly-displaced stomach for one with an hour-glass contraction. In the former the shadow passes at once from an upper chamber into one immediately below, whereas in the latter a long interval elapses, and the distal pouch is then seen to lie to the right of, and not below the proximal.

The chronic pain of gastric ulcer may be simulated in cases of epigastric hernia, gastroptosis, arterio-sclerosis and tabetic crises, and such conditions should always be borne in mind before submitting a case to operation.

**Treatment.**—It is only the chronic ulcer, or that complicated by perforation or intractable hemorrhage, that requires surgical treatment. Until recently it has been usual to regard the operation of gastro-enterostomy as a sovereign remedy for all chronic ulcers ; but this view requires modification. The case most certainly and permanently relieved by this operation is one in which there is definite stenosis of the pylorus, and in which the symptoms are those of this stenosis (*i.e.* emaciation, vomiting, and the decomposition of the stomach contents). But when the ulcer affects the lesser curvature of the stomach, or when the chief symptom is chronic bleeding, then a more efficient remedy consists in an excision of the ulcer, or a resection of that part of the stomach containing it. And the advisability of performing a wide resection rather than a mere short-circuiting operation is strengthened by two considerations : (1) That many of the chronic inflammatory masses are only the early stages of malignant growth ; (2) after gastro-enterostomy there is a tendency for a peptic ulcer to form in the jejunum, and this may cause the same symptoms as those due to the original disease, or it may lead to perforation. It is difficult to compare the results of the two methods, *i.e.* of gastro-enterostomy and resection, because the latter being comparatively new, its technique is not perfected to the same extent as the former, and sufficient time has not elapsed for its lasting results to be confirmed. Payr gives the following figures. Immediate mortality—for gastro-enterostomy, 5 per cent.; for resection (600 cases), 10 to 12 per cent. Ultimate cures—for gastro-enterostomy, 50 to 75 per cent.; for resection, nearly all cases are up to the present fully cured, but sufficient time has not yet elapsed to state definite figures.

In cases associated with hemorrhage it is of the utmost importance to determine whether the causative condition is a recent acute or an old chronic ulcer. In the former case an operation is rarely required. Atropine in full doses has been found to be of great service, because by paralysing the stomach it allows the natural means of arrest to take effect. If the bleeding is from a chronic ulcer, the operative treatment ought not to be delayed, because the longer one waits the worse does the patient's condition become. If the patient is desperately ill, a gastro-enterostomy is the quickest operation, but it is by no means of constant permanent efficacy. Therefore, if possible, the bleeding ulcer ought to be excised, or if it is situated in the pylorus this should be divided after the performance of a gastro-enterostomy, or, best of all, the diseased pylorus should be resected.

After a perforation has been located and dealt with, it is a matter of importance to keep the stomach at rest, and at the same time it is necessary to feed the patient, especially if they are weakened by long illness. In some cases it is possible to perform a gastro-enterostomy after closing the perforation, but this involves danger both from prolongation of the operation and from a diffusion of septic material. In these cases one of two things may be done: (1) A tube is fixed through a small stab wound in the front of the stomach, and thence passed through the pylorus into the duodeum; or (2) a tube is tied into the jejunum, and in either case this tube serves for feeding the patient. Mayo Robson has recently advocated the latter method, both for its simplicity and efficacy.

E. W. Hey Groves.

REFERENCES.

Payr, *Jahresb. für ärztliche Fortbildung*, 1911, ii., Dec., p. 1.
Rowlands, *Brit. M. J.*, 1911, i. 669.
Schmieden, *Arch. f. klin. Chir.*, 1911, xcvi. 253.
Holzknecht, *Jahresb. für ärztliche Fortbildung*, 1911, ii., Aug., p. 89.

Holzknecht, *Deutsche Zeit. f. Chir.*, 1910, cv. 54.

Hertz, *Lancet*, 1911, 1, 1051, 1119, 1187.

Mayo Robson, *Brit. M. J.*, 1912, i. 1.

NOTE.—In the above account, when no special authorities are quoted, they may be found by a reference to Prof. Payr's article, to which is appended a long list of original papers.

